# Di-μ-chlorido-bis­[(2-amino-4-methyl­pyridine-κ*N*)­chloridomercury(II)]

**DOI:** 10.1107/S1600536812039803

**Published:** 2012-09-26

**Authors:** Azadeh Tadjarodi, Keyvan Bijanzad, Behrouz Notash

**Affiliations:** a Department of Chemistry, Iran University of Science and Technology, Tehran 16846-13114, Iran; b Department of Chemistry, Shahid Beheshti University, G. C., Evin, Tehran 1983963113, Iran

## Abstract

In the centrosymmetric dinuclear title compound, [Hg_2_Cl_4_(C_6_H_8_N_2_)_2_], the Hg^II^ ion is four-coordinated by one pyridine N atom from a 2-amino-4-methyl­pyridine ligand, one terminal Cl atom and two bridging Cl atoms. A distorted tetra­hedral geometry is formed around each Hg^II^ ion. The crystal packing is stabilized by intra- and inter­molecular N—H⋯Cl hydrogen bonding. There are also π–π stacking inter­actions in the structure, with centroid-to-centroid distances of 3.594 (6) Å.

## Related literature

For a coordination compound of 2-amino-4-methyl­pyridine, see: Arab Ahmadi *et al.* (2011 ▶). For proton-transfer compounds of 2-amino-4-methyl­pyridine, see: Gharbia *et al.* (2008 ▶); Choudhury *et al.* (2009 ▶); Das *et al.* (2010 ▶); Hemamalini & Fun (2010 ▶); Aghabozorg *et al.* (2011 ▶); Eshtiagh-Hosseini *et al.* (2010 ▶). For mixed-ligand complexes of 2-amino-4-methyl­pyridine, see: Zhang *et al.* (2008 ▶); Castillo *et al.* (2001 ▶); Yenikaya *et al.* (2011 ▶). For similar structures, see: Baul *et al.* (2004 ▶).
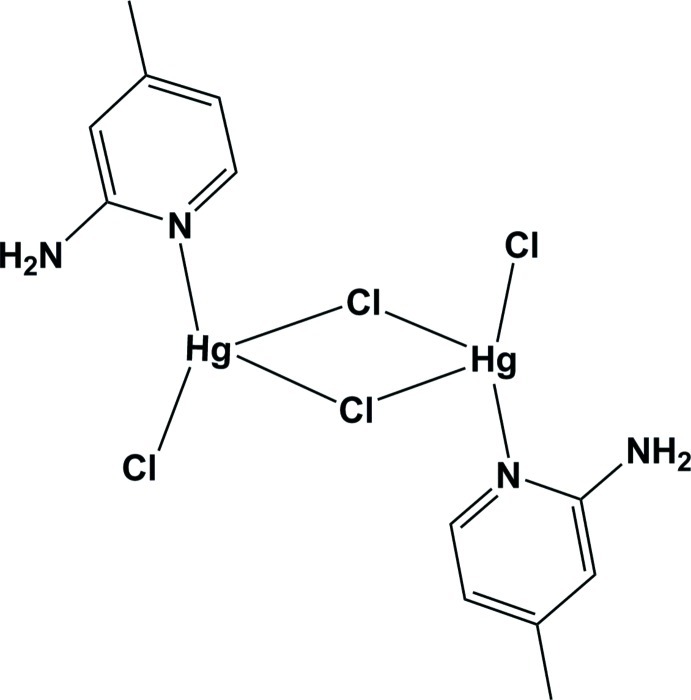


## Experimental

### 

#### Crystal data

[Hg_2_Cl_4_(C_6_H_8_N_2_)_2_]
*M* *_r_* = 759.27
Monoclinic, 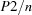

*a* = 7.1777 (14) Å
*b* = 9.1672 (18) Å
*c* = 14.546 (3) Å
β = 101.92 (3)°
*V* = 936.5 (3) Å^3^
*Z* = 2
Mo *K*α radiation
μ = 16.94 mm^−1^
*T* = 120 K
0.25 × 0.25 × 0.20 mm

#### Data collection

Stoe IPDS 2T diffractometer
Absorption correction: numerical (shape of crystal determined optically; *X-RED* and *X-SHAPE*, Stoe & Cie, 2005 ▶) *T*_min_ = 0.101, *T*_max_ = 0.133
6495 measured reflections
2507 independent reflections
2019 reflections with *I* > 2σ(*I*)
*R*_int_ = 0.090

#### Refinement

*R*[*F* ^2^ > 2σ(*F* ^2^)] = 0.046
*wR*(*F* ^2^) = 0.111
*S* = 1.00
2507 reflections
109 parameters
2 restraints
H atoms treated by a mixture of independent and constrained refinement
Δρ_max_ = 2.35 e Å^−3^
Δρ_min_ = −2.80 e Å^−3^

### 

Data collection: *X-AREA* (Stoe & Cie, 2005 ▶); cell refinement: *X-AREA*; data reduction: *X-AREA*; program(s) used to solve structure: *SHELXS97* (Sheldrick, 2008 ▶); program(s) used to refine structure: *SHELXL97* (Sheldrick, 2008 ▶); molecular graphics: *ORTEP-3 for Windows* (Farrugia, 1997 ▶); software used to prepare material for publication: *WinGX* (Farrugia, 1999 ▶).

## Supplementary Material

Crystal structure: contains datablock(s) I, global. DOI: 10.1107/S1600536812039803/bt6826sup1.cif

Structure factors: contains datablock(s) I. DOI: 10.1107/S1600536812039803/bt6826Isup2.hkl

Additional supplementary materials:  crystallographic information; 3D view; checkCIF report

## Figures and Tables

**Table 1:** Hydrogen-bond geometry (Å, °)

*D*—H⋯*A*	*D*—H	H⋯*A*	*D*⋯*A*	*D*—H⋯*A*
N2—H2*B*⋯Cl2^i^	0.87 (2)	2.62 (7)	3.380 (8)	146 (11)
N2—H2*A*⋯Cl2^ii^	0.87 (2)	2.68 (11)	3.379 (9)	139 (13)

Symmetry codes: (i) 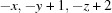; (ii) 
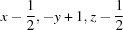
.

**Fig. 1.**
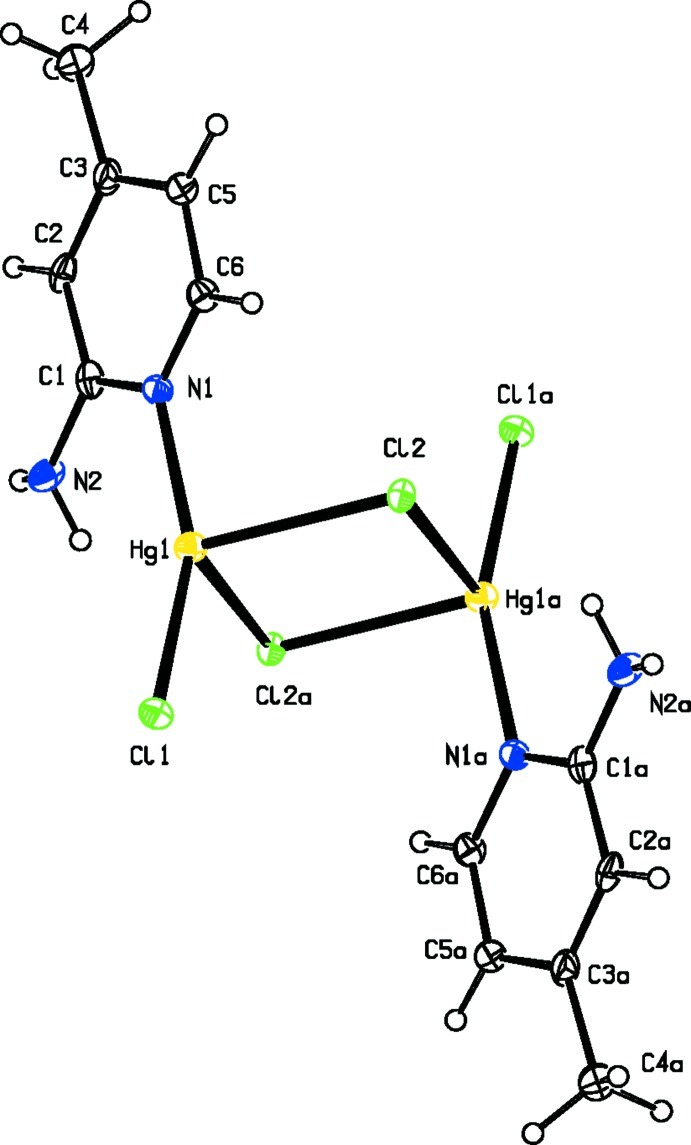
The molecular structure of [Hg2(C6H8N2)2Cl4] with displacement ellipsoids drawn at 30% probability level.

**Fig. 2.**
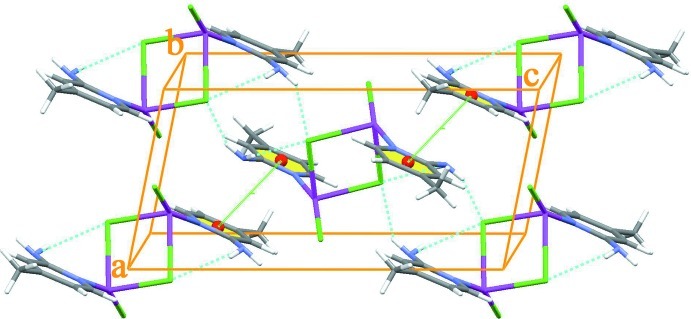
The packing diagram of the title compound showing hydrogen bonding as blue dashed lines and π–π stacking.

## supplementary crystallographic information

### Comment

To the best of our knowledge, coordination chemistry of 2-amino-4-methylpyridine has not been explored sufficiently. It has the potential to coordinate to metals through the N atom of the pyridyl group (Arab Ahmadi *et al.*, 2011).

Contrary to other derivatives of aminopicolines, less work has been done on the synthesis of the complexes merely containing 2-amino-4-methylpyridine and most of the work is focused on its mixed ligand complexes (Zhang *et al.*, 2008; Castillo *et al.*, 2001; Yenikaya *et al.*, 2011) or proton transfer compounds (Gharbia *et al.*, 2008; Choudhury *et al.*, 2009; Das *et al.*, 2010; Hemamalini & Fun, 2010; Aghabozorg *et al.*, 2011; Eshtiagh-Hosseini *et al.*, 2010).

Herein, we report the synthesis and crystal structure of the title compound, [Hg_2_(C_6_H_8_N_2_)_2_Cl_4_], which is a dimeric complex. The centrosymmetric structure is made up of two Hg atoms, each coordinated by a terminal chloride atom and a nitrogen pyridyl ligand and bridged by chloride atoms. A distorted tetrahedral geometry is formed around each metal center (Fig. 1). The asymmetric unit contains one-half of a molecule since the whole molecule lies across a crystallographic inversion center. Bridging chloride atoms along with amino groups take part in intra- and intermolecular N—H··· Cl hydrogen bonds (Fig. 2 and Table 1). There is also π–π stacking in the structure of title compound with centroid–centroid distance of 3.594 (6) Å (Fig. 2).

### Experimental

A solution of 2-amino-4-methylpyridine (1 mmol) in methanol was added to a methanolic solution of HgCl_2_ (1 mmol) and stirred for 20 min at 50°C. Slow evaporation of the resulting solution gave colorless crystals suitable for X-ray analysis.

### Refinement

H atoms attached to N atoms were found in difference Fourier map. They were refined with distance restraints of N—H 0.87 (2). H atoms attached to C atoms were positioned geometrically and refined as riding atoms, with C—H = 0.93 Å (CH), with C—H = 0.96 Å (CH_3_), and *U*_iso_(H) = 1.2*U*_eq_(C) or 1.5*U*_eq_(C_methyl_).

### Crystal data

**Table:**

C_12_H_16_Cl_4_Hg_2_N_4_	*F*(000) = 688
*M**_r_* = 759.27	*D*_x_ = 2.693 Mg m^−^^3^
Monoclinic, *P*2/*n*	Mo *K*α radiation, λ = 0.71073 Å
Hall symbol: -P 2yac	Cell parameters from 2507 reflections
*a* = 7.1777 (14) Å	θ = 2.6–29.1°
*b* = 9.1672 (18) Å	µ = 16.94 mm^−^^1^
*c* = 14.546 (3) Å	*T* = 120 K
β = 101.92 (3)°	Block, colorless
*V* = 936.5 (3) Å^3^	0.25 × 0.25 × 0.20 mm
*Z* = 2	

### Data collection

**Table:**

Stoe IPDS 2T diffractometer	2507 independent reflections
Radiation source: fine-focus sealed tube	2019 reflections with *I* > 2σ(*I*)
Graphite monochromator	*R*_int_ = 0.090
Detector resolution: 0.15 mm pixels mm^-1^	θ_max_ = 29.1°, θ_min_ = 2.6°
rotation method scans	*h* = −9→9
Absorption correction: numerical shape of crystal determined optically	*k* = −12→10
*T*_min_ = 0.101, *T*_max_ = 0.133	*l* = −19→19
6495 measured reflections	

### Refinement

**Table:**

Refinement on *F*^2^	Primary atom site location: structure-invariant direct methods
Least-squares matrix: full	Secondary atom site location: difference Fourier map
*R*[*F*^2^ > 2σ(*F*^2^)] = 0.046	Hydrogen site location: inferred from neighbouring sites
*wR*(*F*^2^) = 0.111	H atoms treated by a mixture of independent and constrained refinement
*S* = 1.00	*w* = 1/[σ^2^(*F*_o_^2^) + (0.0604*P*)^2^] where *P* = (*F*_o_^2^ + 2*F*_c_^2^)/3
2507 reflections	(Δ/σ)_max_ < 0.001
109 parameters	Δρ_max_ = 2.35 e Å^−^^3^
2 restraints	Δρ_min_ = −2.80 e Å^−^^3^

### Special details

**Table:**

Geometry. All e.s.d.'s (except the e.s.d. in the dihedral angle between two l.s. planes) are estimated using the full covariance matrix. The cell e.s.d.'s are taken into account individually in the estimation of e.s.d.'s in distances, angles and torsion angles; correlations between e.s.d.'s in cell parameters are only used when they are defined by crystal symmetry. An approximate (isotropic) treatment of cell e.s.d.'s is used for estimating e.s.d.'s involving l.s. planes.
Refinement. Refinement of *F*^2^ against ALL reflections. The weighted *R*-factor *wR* and goodness of fit *S* are based on *F*^2^, conventional *R*-factors *R* are based on *F*, with *F* set to zero for negative *F*^2^. The threshold expression of *F*^2^ > σ(*F*^2^) is used only for calculating *R*-factors(gt) *etc*. and is not relevant to the choice of reflections for refinement. *R*-factors based on *F*^2^ are statistically about twice as large as those based on *F*, and *R*-factors based on ALL data will be even larger.

### Fractional atomic coordinates and isotropic or equivalent isotropic displacement parameters (Å^2^)

**Table:**

	*x*	*y*	*z*	*U*_iso_*/*U*_eq_	
Hg1	0.21238 (5)	0.47797 (4)	0.94011 (2)	0.02164 (12)	
Cl1	0.3952 (3)	0.6882 (3)	0.98493 (15)	0.0237 (4)	
Cl2	0.1382 (3)	0.3856 (2)	1.10331 (13)	0.0205 (4)	
N1	0.1135 (11)	0.2841 (9)	0.8633 (5)	0.0210 (15)	
N2	0.0000 (13)	0.4145 (10)	0.7272 (5)	0.0278 (18)	
C1	0.0312 (12)	0.2842 (11)	0.7714 (6)	0.0221 (18)	
C2	−0.0152 (13)	0.1524 (12)	0.7224 (6)	0.025 (2)	
H2	−0.0709	0.1541	0.6572	0.030*	
C3	0.0199 (16)	0.0208 (12)	0.7685 (7)	0.031 (2)	
C4	−0.022 (2)	−0.1198 (14)	0.7162 (8)	0.046 (3)	
H4A	−0.1242	−0.1048	0.6610	0.068*	
H4B	−0.0612	−0.1931	0.7573	0.068*	
H4C	0.0930	−0.1536	0.6959	0.068*	
C5	0.0993 (15)	0.0221 (12)	0.8661 (7)	0.028 (2)	
H5	0.1206	−0.0662	0.9009	0.034*	
C6	0.1444 (14)	0.1542 (11)	0.9089 (6)	0.0241 (18)	
H6	0.2003	0.1550	0.9741	0.029*	
H2A	−0.080 (14)	0.429 (19)	0.675 (5)	0.06 (4)*	
H2B	−0.028 (19)	0.495 (7)	0.753 (9)	0.03 (3)*	

### Atomic displacement parameters (Å^2^)

**Table:**

	*U*^11^	*U*^22^	*U*^33^	*U*^12^	*U*^13^	*U*^23^
Hg1	0.02222 (18)	0.0215 (2)	0.01994 (17)	−0.00052 (14)	0.00134 (11)	−0.00125 (13)
Cl1	0.0237 (10)	0.0218 (12)	0.0245 (10)	−0.0020 (8)	0.0025 (8)	0.0015 (8)
Cl2	0.0194 (9)	0.0248 (11)	0.0177 (8)	0.0030 (8)	0.0047 (7)	0.0008 (7)
N1	0.021 (4)	0.020 (4)	0.022 (3)	−0.003 (3)	0.005 (3)	−0.001 (3)
N2	0.040 (5)	0.026 (5)	0.016 (3)	0.010 (4)	0.001 (3)	−0.001 (3)
C1	0.010 (4)	0.033 (5)	0.024 (4)	0.001 (3)	0.005 (3)	0.003 (3)
C2	0.020 (4)	0.038 (6)	0.017 (4)	−0.004 (4)	0.006 (3)	−0.010 (4)
C3	0.038 (6)	0.033 (6)	0.026 (4)	−0.015 (4)	0.017 (4)	−0.012 (4)
C4	0.073 (9)	0.036 (7)	0.033 (5)	−0.030 (6)	0.023 (6)	−0.022 (5)
C5	0.033 (5)	0.023 (5)	0.031 (5)	0.003 (4)	0.014 (4)	0.002 (4)
C6	0.025 (5)	0.026 (5)	0.019 (4)	0.001 (4)	0.002 (3)	0.002 (3)

### Geometric parameters (Å, º)

**Table:**

Hg1—N1	2.141 (8)	C2—C3	1.377 (16)
Hg1—Cl1	2.347 (2)	C2—H2	0.9500
Hg1—Cl2	2.6755 (19)	C3—C5	1.417 (16)
Hg1—Cl2^i^	2.763 (2)	C3—C4	1.496 (14)
Cl2—Hg1^i^	2.763 (2)	C4—H4A	0.9800
N1—C1	1.345 (12)	C4—H4B	0.9800
N1—C6	1.359 (13)	C4—H4C	0.9800
N2—C1	1.353 (13)	C5—C6	1.369 (15)
N2—H2A	0.87 (2)	C5—H5	0.9500
N2—H2B	0.87 (2)	C6—H6	0.9500
C1—C2	1.407 (14)		
			
N1—Hg1—Cl1	158.87 (19)	C3—C2—H2	119.8
N1—Hg1—Cl2	95.45 (19)	C1—C2—H2	119.8
Cl1—Hg1—Cl2	102.46 (7)	C2—C3—C5	118.4 (9)
N1—Hg1—Cl2^i^	93.9 (2)	C2—C3—C4	120.6 (10)
Cl1—Hg1—Cl2^i^	97.04 (8)	C5—C3—C4	120.9 (11)
Cl2—Hg1—Cl2^i^	90.41 (7)	C3—C4—H4A	109.5
Hg1—Cl2—Hg1^i^	89.59 (7)	C3—C4—H4B	109.5
C1—N1—C6	118.7 (9)	H4A—C4—H4B	109.5
C1—N1—Hg1	123.2 (7)	C3—C4—H4C	109.5
C6—N1—Hg1	118.0 (6)	H4A—C4—H4C	109.5
C1—N2—H2A	125 (10)	H4B—C4—H4C	109.5
C1—N2—H2B	125 (9)	C6—C5—C3	118.1 (10)
H2A—N2—H2B	94 (10)	C6—C5—H5	121.0
N1—C1—N2	117.9 (9)	C3—C5—H5	121.0
N1—C1—C2	120.8 (9)	N1—C6—C5	123.6 (9)
N2—C1—C2	121.3 (8)	N1—C6—H6	118.2
C3—C2—C1	120.3 (8)	C5—C6—H6	118.2
			
N1—Hg1—Cl2—Hg1^i^	−94.0 (2)	C6—N1—C1—C2	−2.5 (12)
Cl1—Hg1—Cl2—Hg1^i^	97.30 (8)	Hg1—N1—C1—C2	174.1 (6)
Cl2^i^—Hg1—Cl2—Hg1^i^	0.0	N1—C1—C2—C3	1.2 (12)
Cl1—Hg1—N1—C1	−64.4 (10)	N2—C1—C2—C3	179.2 (9)
Cl2—Hg1—N1—C1	147.6 (6)	C1—C2—C3—C5	1.4 (14)
Cl2^i^—Hg1—N1—C1	56.8 (6)	C1—C2—C3—C4	−177.6 (9)
Cl1—Hg1—N1—C6	112.2 (7)	C2—C3—C5—C6	−2.6 (15)
Cl2—Hg1—N1—C6	−35.8 (6)	C4—C3—C5—C6	176.3 (9)
Cl2^i^—Hg1—N1—C6	−126.6 (6)	C1—N1—C6—C5	1.2 (13)
C6—N1—C1—N2	179.4 (8)	Hg1—N1—C6—C5	−175.6 (7)
Hg1—N1—C1—N2	−4.0 (11)	C3—C5—C6—N1	1.4 (15)

Symmetry code: (i) −*x*, −*y*+1, −*z*+2.

### Hydrogen-bond geometry (Å, º)

**Table:**

*D*—H···*A*	*D*—H	H···*A*	*D*···*A*	*D*—H···*A*
N2—H2*B*···Cl2^i^	0.87 (2)	2.62 (7)	3.380 (8)	146 (11)
N2—H2*A*···Cl2^ii^	0.87 (2)	2.68 (11)	3.379 (9)	139 (13)

Symmetry codes: (i) −*x*, −*y*+1, −*z*+2; (ii) *x*−1/2, −*y*+1, *z*−1/2.
